# Supplementary material for: Functional and structural analyses reveal that a dual domain sialidase protects bacteria from complement killing through desialylation of complement factors
Source: PLoS Pathog. 2023 Sep 25;19(9):e1011674. doi: 10.1371/journal.ppat.1011674 (PMC10553830; doi:10.1371/journal.ppat.1011674)
Supplement: S1 Table — (PDF) [file ppat.1011674.s001.pdf]

S1 Table. Primers used in this study

Primer	Orientation	Sequence (5' - 3')	Description
P1	F	GCGGCGGCGATTCTGAAGACCGCG	Primer to mutate PG0352:YRIP to AAAA
P2	R	CGCCGCGTTACGGCTGCCACCATC	Primer to mutate PG0352:YRIP to AAAA
P3	F	CACCATGGCGCTGTTTGTTCCGGGTGA	Overexpression of PG0352CT in pET200
P4	R	TTATTGACGCACGTCGAACAGGTCGT	Overexpression of PG0352CT in pET200
P5	F	CACCATGCAGGAAGTCACTATGTGGG	Overexpression of PGD in pET101
P6	R	CTCATACCCCCTATACAGACGAC	Overexpression or PGD in pET101
