# Supplementary material for: Functional and structural analyses reveal that a dual domain sialidase protects bacteria from complement killing through desialylation of complement factors
Source: PLoS Pathog. 2023 Sep 25;19(9):e1011674. doi: 10.1371/journal.ppat.1011674 (PMC10553830; doi:10.1371/journal.ppat.1011674)
Supplement: S2 Table — (PDF) [file ppat.1011674.s002.pdf]

S2 Table. MS/MS Spectra for nano-LC/MS/MS for human C1q before and after the treatment of PG0352

Peptide Sequences	Identified Glycans	Peptide Sequences	Identified Glycans
R.RNPPM[+15.99492]GGNVVIFDTVITNQEEPYQN[+2789.99933]HSGR.F	HexNAc(6)Hex(7)Fuc(1)NeuAc(1)	R.RNPPM[+15.99492]GGNVVIFDTVITNQEEPYQN[+2498.90391]HSGR.F	HexNAc(6)Hex(7)Fuc(1)
R.RNPPM[+15.99492]GGNVVIFDTVITNQEEPYQN[+2717.97820]HSGR.F	HexNAc(7)Hex(8)	R.RNPPM[+15.99492]GGNVVIFDTVITNQEEPYQN[+2498.90391]HSGR.F	HexNAc(6)Hex(7)Fuc(1)
R.RNPPM[+15.99492]GGNVVIFDTVITNQEEPYQN[+2715.96255]HSGR.F	HexNAc(5)Hex(6)Fuc(1)NeuAc(2)	R.RNPPM[+15.99492]GGNVVIFDTVITNQEEPYQN[+2352.84600]HSGR.F	HexNAc(6)Hex(7)
R.RNPPM[+15.99492]GGNVVIFDTVITNQEEPYQN[+2481.88859]HSGR.F	HexNAc(6)Hex(6)NeuAc(1)	R.RNPPM[+15.99492]GGNVVIFDTVITNQEEPYQN[+2279.82962]HSGR.F	HexNAc(5)Hex(6)Fuc(2)
R.RNPPM[+15.99492]GGNVVIFDTVITNQEEPYQN[+2434.89910]HSGR.F	HexNAc(8)Hex(5)	R.RNPPM[+15.99492]GGNVVIFDTVITNQEEPYQN[+2158.80335]HSGR.F	HexNAc(6)Hex(4)Fuc(2)
R.RNPPM[+15.99492]GGNVVIFDTVITNQEEPYQN[+2432.88345]HSGR.F	HexNAc(6)Hex(3)Fuc(1)NeuAc(2)	R.RNPPM[+15.99492]GGNVVIFDTVITNQEEPYQN[+2149.76663]HSGR.F	HexNAc(5)Hex(7)
R.RNPPM[+15.99492]GGNVVIFDTVITNQEEPYQN[+2425.88753]HSGR.F	HexNAc(5)Hex(6)Fuc(3)	R.RNPPM[+15.99492]GGNVVIFDTVITNQEEPYQN[+2142.80843]HSGR.F	HexNAc(6)Hex(3)Fuc(3)
R.RNPPM[+15.99492]GGNVVIFDTVITNQEEPYQN[+2424.86713]HSGR.F	HexNAc(5)Hex(6)Fuc(1)NeuAc(1)	R.RNPPM[+15.99492]GGNVVIFDTVITNQEEPYQN[+2133.77171]HSGR.F	HexNAc(5)Hex(6)Fuc(1)
R.RNPPM[+15.99492]GGNVVIFDTVITNQEEPYQN[+2393.87255]HSGR.F	HexNAc(7)Hex(6)	R.RNPPM[+15.99492]GGNVVIFDTVITNQEEPYQN[+2018.70838]HSGR.F	HexNAc(3)Hex(6)Fuc(1)NeuAc(1)
R.RNPPM[+15.99492]GGNVVIFDTVITNQEEPYQN[+2361.88272]HSGR.F	HexNAc(7)Hex(4)Fuc(2)	R.RNPPM[+15.99492]GGNVVIFDTVITNQEEPYQN[+2012.74544]HSGR.F	HexNAc(6)Hex(4)Fuc(1)
R.RNPPM[+15.99492]GGNVVIFDTVITNQEEPYQN[+2351.85075]HSGR.F	HexNAc(4)Hex(5)Fuc(3)NeuAc(1)	R.RNPPM[+15.99492]GGNVVIFDTVITNQEEPYQN[+1987.71380]HSGR.F	HexNAc(5)Hex(6)
R.RNPPM[+15.99492]GGNVVIFDTVITNQEEPYQN[+2350.83035]HSGR.F	HexNAc(4)Hex(5)Fuc(1)NeuAc(2)	R.RNPPM[+15.99492]GGNVVIFDTVITNQEEPYQN[+1971.71889]HSGR.F	HexNAc(5)Hex(5)Fuc(1)
R.RNPPM[+15.99492]GGNVVIFDTVITNQEEPYQN[+2279.82962]HSGR.F	HexNAc(5)Hex(6)Fuc(2)	R.RNPPM[+15.99492]GGNVVIFDTVITNQEEPYQN[+1914.69743]HSGR.F	HexNAc(4)Hex(5)Fuc(2)
R.RNPPM[+15.99492]GGNVVIFDTVITNQEEPYQN[+2278.80922]HSGR.F	HexNAc(5)Hex(6)NeuAc(1)	R.RNPPM[+15.99492]GGNVVIFDTVITNQEEPYQN[+1825.66098]HSGR.F	HexNAc(5)Hex(5)
R.RNPPM[+15.99492]GGNVVIFDTVITNQEEPYQN[+2277.78882]HSGR.F	HexNAc(5)Hex(4)NeuGc(2)	R.RNPPM[+15.99492]GGNVVIFDTVITNQEEPYQN[+1784.63443]HSGR.F	HexNAc(4)Hex(6)
R.RNPPM[+15.99492]GGNVVIFDTVITNQEEPYQN[+2205.79284]HSGR.F	HexNAc(4)Hex(5)Fuc(2)NeuAc(1)	R.RNPPM[+15.99492]GGNVVIFDTVITNQEEPYQN[+1768.63952]HSGR.F	HexNAc(4)Hex(5)Fuc(1)
R.RNPPM[+15.99492]GGNVVIFDTVITNQEEPYQN[+2204.77244]HSGR.F	HexNAc(4)Hex(5)NeuAc(2)	R.RNPPM[+15.99492]GGNVVIFDTVITNQEEPYQN[+1622.58161]HSGR.F	HexNAc(4)Hex(5)
R.RNPPM[+15.99492]GGNVVIFDTVITNQEEPYQN[+2133.77171]HSGR.F	HexNAc(5)Hex(6)Fuc(1)	R.RNPPM[+15.99492]GGNVVIFDTVITNQEEPYQN[+1606.58669]HSGR.F	HexNAc(4)Hex(4)Fuc(1)
R.RNPPM[+15.99492]GGNVVIFDTVITNQEEPYQN[+2116.75640]HSGR.F	HexNAc(5)Hex(5)NeuAc(1)	R.NPPM[+15.99492]GGNVVIFDTVITNQEEPYQN[+2539.93046]HSGR.F	HexNAc(7)Hex(6)Fuc(1)
R.RNPPM[+15.99492]GGNVVIFDTVITNQEEPYQN[+2100.76148]HSGR.F	HexNAc(5)Hex(4)Fuc(1)NeuAc(1)	R.NPPM[+15.99492]GGNVVIFDTVITNQEEPYQN[+2279.82962]HSGR.F	HexNAc(5)Hex(6)Fuc(2)
R.RNPPM[+15.99492]GGNVVIFDTVITNQEEPYQN[+2091.72476]HSGR.F	HexNAc(4)Hex(6)NeuGc(1)	R.NPPM[+15.99492]GGNVVIFDTVITNQEEPYQN[+2263.83471]HSGR.F	HexNAc(5)Hex(5)Fuc(3)
R.RNPPM[+15.99492]GGNVVIFDTVITNQEEPYQN[+2075.72985]HSGR.F	HexNAc(4)Hex(6)NeuAc(1)	R.NPPM[+15.99492]GGNVVIFDTVITNQEEPYQN[+2215.82481]HSGR.F	HexNAc(7)Hex(4)Fuc(1)
R.RNPPM[+15.99492]GGNVVIFDTVITNQEEPYQN[+2059.73493]HSGR.F	HexNAc(4)Hex(5)Fuc(1)NeuAc(1)	R.NPPM[+15.99492]GGNVVIFDTVITNQEEPYQN[+2158.80335]HSGR.F	HexNAc(6)Hex(4)Fuc(2)
R.RNPPM[+15.99492]GGNVVIFDTVITNQEEPYQN[+1987.71380]HSGR.F	HexNAc(5)Hex(6)	R.NPPM[+15.99492]GGNVVIFDTVITNQEEPYQN[+2149.76663]HSGR.F	HexNAc(5)Hex(7)
R.RNPPM[+15.99492]GGNVVIFDTVITNQEEPYQN[+1914.69743]HSGR.F	HexNAc(4)Hex(5)Fuc(2)	R.NPPM[+15.99492]GGNVVIFDTVITNQEEPYQN[+2133.77171]HSGR.F	HexNAc(5)Hex(6)Fuc(1)
R.RNPPM[+15.99492]GGNVVIFDTVITNQEEPYQN[+1913.67702]HSGR.F	HexNAc(4)Hex(5)NeuAc(1)	R.NPPM[+15.99492]GGNVVIFDTVITNQEEPYQN[+2069.76690]HSGR.F	HexNAc(7)Hex(4)
R.RNPPM[+15.99492]GGNVVIFDTVITNQEEPYQN[+1897.68211]HSGR.F	HexNAc(4)Hex(4)Fuc(1)NeuAc(1)	R.NPPM[+15.99492]GGNVVIFDTVITNQEEPYQN[+2059.73493]HSGR.F	HexNAc(4)Hex(5)Fuc(1)NeuAc(1)
R.RNPPM[+15.99492]GGNVVIFDTVITNQEEPYQN[+1768.63952]HSGR.F	HexNAc(4)Hex(5)Fuc(1)	R.NPPM[+15.99492]GGNVVIFDTVITNQEEPYQN[+1987.71380]HSGR.F	HexNAc(5)Hex(6)
R.RNPPM[+15.99492]GGNVVIFDTVITNQEEPYQN[+1622.58161]HSGR.F	HexNAc(4)Hex(5)	R.NPPM[+15.99492]GGNVVIFDTVITNQEEPYQN[+1971.71889]HSGR.F	HexNAc(5)Hex(5)Fuc(1)
R.NPPM[+15.99492]GGNVVIFDTVITNQEEPYQN[+2715.96255]HSGR.F	HexNAc(5)Hex(6)Fuc(1)NeuAc(2)	R.NPPM[+15.99492]GGNVVIFDTVITNQEEPYQN[+1914.69743]HSGR.F	HexNAc(4)Hex(5)Fuc(2)
R.NPPM[+15.99492]GGNVVIFDTVITNQEEPYQN[+2570.92504]HSGR.F	HexNAc(5)Hex(6)Fuc(2)NeuAc(1)	R.NPPM[+15.99492]GGNVVIFDTVITNQEEPYQN[+1913.67702]HSGR.F	HexNAc(4)Hex(5)NeuAc(1)
R.NPPM[+15.99492]GGNVVIFDTVITNQEEPYQN[+2424.86713]HSGR.F	HexNAc(5)Hex(6)Fuc(1)NeuAc(1)	R.NPPM[+15.99492]GGNVVIFDTVITNQEEPYQN[+1825.66098]HSGR.F	HexNAc(5)Hex(5)
R.NPPM[+15.99492]GGNVVIFDTVITNQEEPYQN[+2366.82526]HSGR.F	HexNAc(4)Hex(5)Fuc(1)NeuAc(1)NeuGc(1)	R.NPPM[+15.99492]GGNVVIFDTVITNQEEPYQN[+1784.63443]HSGR.F	HexNAc(4)Hex(6)
R.NPPM[+15.99492]GGNVVIFDTVITNQEEPYQN[+2351.85075]HSGR.F	HexNAc(4)Hex(5)Fuc(3)NeuAc(1)	R.NPPM[+15.99492]GGNVVIFDTVITNQEEPYQN[+1768.63952]HSGR.F	HexNAc(4)Hex(5)Fuc(1)
R.NPPM[+15.99492]GGNVVIFDTVITNQEEPYQN[+2350.83035]HSGR.F	HexNAc(4)Hex(5)Fuc(1)NeuAc(2)	R.NPPM[+15.99492]GGNVVIFDTVITNQEEPYQN[+1622.58161]HSGR.F	HexNAc(4)Hex(5)
R.NPPM[+15.99492]GGNVVIFDTVITNQEEPYQN[+2278.80922]HSGR.F	HexNAc(5)Hex(6)NeuAc(1)		
R.NPPM[+15.99492]GGNVVIFDTVITNQEEPYQN[+2206.81324]HSGR.F	HexNAc(4)Hex(5)Fuc(4)		
R.NPPM[+15.99492]GGNVVIFDTVITNQEEPYQN[+2205.79284]HSGR.F	HexNAc(4)Hex(5)Fuc(2)NeuAc(1)		
R.NPPM[+15.99492]GGNVVIFDTVITNQEEPYQN[+2204.77244]HSGR.F	HexNAc(4)Hex(5)NeuAc(2)		
R.NPPM[+15.99492]GGNVVIFDTVITNQEEPYQN[+2133.77171]HSGR.F	HexNAc(5)Hex(6)Fuc(1)		
R.NPPM[+15.99492]GGNVVIFDTVITNQEEPYQN[+2116.75640]HSGR.F	HexNAc(5)Hex(5)NeuAc(1)		
R.NPPM[+15.99492]GGNVVIFDTVITNQEEPYQN[+2059.73493]HSGR.F	HexNAc(4)Hex(5)Fuc(1)NeuAc(1)		
R.NPPM[+15.99492]GGNVVIFDTVITNQEEPYQN[+1914.69743]HSGR.F	HexNAc(4)Hex(5)Fuc(2)		
R.NPPM[+15.99492]GGNVVIFDTVITNQEEPYQN[+1913.67702]HSGR.F	HexNAc(4)Hex(5)NeuAc(1)		
R.NPPM[+15.99492]GGNVVIFDTVITNQEEPYQN[+1897.68211]HSGR.F	HexNAc(4)Hex(4)Fuc(1)NeuAc(1)		
R.NPPM[+15.99492]GGNVVIFDTVITNQEEPYQN[+1768.63952]HSGR.F	HexNAc(4)Hex(5)Fuc(1)		
R.NPPM[+15.99492]GGNVVIFDTVITNQEEPYQN[+1622.58161]HSGR.F	HexNAc(4)Hex(5)		

Note: left two panels: glycopeptides and glycans detected in C1q without PG0352 treatment; right two panels: glycopeptides and glycans detected in C1q treated with PG0352
