# Supplementary material for: Functional and structural analyses reveal that a dual domain sialidase protects bacteria from complement killing through desialylation of complement factors
Source: PLoS Pathog. 2023 Sep 25;19(9):e1011674. doi: 10.1371/journal.ppat.1011674 (PMC10553830; doi:10.1371/journal.ppat.1011674)
Supplement: S3 Table — (PDF) [file ppat.1011674.s003.pdf]

S3 Table. MS/MS Spectra for nano-LC/MS/MS for human C1q before and after the treatment of PG0352

Peptide Sequences	Identified Glycans	Peptide Sequences	Identified Glycans
R.RNPPM[+15.99492]GGNVVIFDTVITNQEEPYQN[+2475.92565]HSGR.F	HexNAc(9)Hex(4)	R.RNPPM[+15.99492]GGNVVIFDTVITNQEEPYQN[+2133.77171]HSGR.F	HexNAc(5)Hex(6)Fuc(1)
R.RNPPM[+15.99492]GGNVVIFDTVITNQEEPYQN[+2465.89368]HSGR.F	HexNAc(6)Hex(5)Fuc(1)NeuAc(1)	R.RNPPM[+15.99492]GGNVVIFDTVITNQEEPYQN[+1987.71380]HSGR.F	HexNAc(5)Hex(6)
R.RNPPM[+15.99492]GGNVVIFDTVITNQEEPYQN[+2382.82018]HSGR.F	HexNAc(4)Hex(5)Fuc(1)NeuGc(2)	R.RNPPM[+15.99492]GGNVVIFDTVITNQEEPYQN[+1914.69743]HSGR.F	HexNAc(4)Hex(5)Fuc(2)
R.RNPPM[+15.99492]GGNVVIFDTVITNQEEPYQN[+2361.88272]HSGR.F	HexNAc(7)Hex(4)Fuc(2)	R.RNPPM[+15.99492]GGNVVIFDTVITNQEEPYQN[+1913.67702]HSGR.F	HexNAc(4)Hex(5)NeuAc(1)
R.RNPPM[+15.99492]GGNVVIFDTVITNQEEPYQN[+2351.85075]HSGR.F	HexNAc(4)Hex(5)Fuc(3)NeuAc(1)	R.RNPPM[+15.99492]GGNVVIFDTVITNQEEPYQN[+1768.63952]HSGR.F	HexNAc(4)Hex(5)Fuc(1)
R.RNPPM[+15.99492]GGNVVIFDTVITNQEEPYQN[+2350.83035]HSGR.F	HexNAc(4)Hex(5)Fuc(1)NeuAc(2)	R.RNPPM[+15.99492]GGNVVIFDTVITNQEEPYQN[+1622.58161]HSGR.F	HexNAc(4)Hex(5)
R.RNPPM[+15.99492]GGNVVIFDTVITNQEEPYQN[+2279.82962]HSGR.F	HexNAc(5)Hex(6)Fuc(2)	R.NPPM[+15.99492]GGNVVIFDTVITNQEEPYQN[+2158.80335]HSGR.F	HexNAc(6)Hex(4)Fuc(2)
R.RNPPM[+15.99492]GGNVVIFDTVITNQEEPYQN[+2278.80922]HSGR.F	HexNAc(5)Hex(6)NeuAc(1)	R.NPPM[+15.99492]GGNVVIFDTVITNQEEPYQN[+2133.77171]HSGR.F	HexNAc(5)Hex(6)Fuc(1)
R.RNPPM[+15.99492]GGNVVIFDTVITNQEEPYQN[+2205.79284]HSGR.F	HexNAc(4)Hex(5)Fuc(2)NeuAc(1)	R.NPPM[+15.99492]GGNVVIFDTVITNQEEPYQN[+1987.71380]HSGR.F	HexNAc(5)Hex(6)
R.RNPPM[+15.99492]GGNVVIFDTVITNQEEPYQN[+2204.77244]HSGR.F	HexNAc(4)Hex(5)NeuAc(2)	R.NPPM[+15.99492]GGNVVIFDTVITNQEEPYQN[+1768.63952]HSGR.F	HexNAc(4)Hex(5)Fuc(1)
R.RNPPM[+15.99492]GGNVVIFDTVITNQEEPYQN[+2060.75533]HSGR.F	HexNAc(4)Hex(5)Fuc(3)	R.NPPM[+15.99492]GGNVVIFDTVITNQEEPYQN[+1622.58161]HSGR.F	HexNAc(4)Hex(5)
R.RNPPM[+15.99492]GGNVVIFDTVITNQEEPYQN[+2059.73493]HSGR.F	HexNAc(4)Hex(5)Fuc(1)NeuAc(1)	R.RNPPMGGNVVIFDTVITNQEEPYQN[+1768.63952]HSGR.F	HexNAc(4)Hex(5)Fuc(1)
R.RNPPM[+15.99492]GGNVVIFDTVITNQEEPYQN[+1914.69743]HSGR.F	HexNAc(4)Hex(5)Fuc(2)	R.NPPMGGNVVIFDTVITNQEEPYQN[+1768.63952]HSGR.F	HexNAc(4)Hex(5)Fuc(1)
R.RNPPM[+15.99492]GGNVVIFDTVITNQEEPYQN[+1913.67702]HSGR.F	HexNAc(4)Hex(5)NeuAc(1)		
R.RNPPM[+15.99492]GGNVVIFDTVITNQEEPYQN[+1768.63952]HSGR.F	HexNAc(4)Hex(5)Fuc(1)		
R.NPPM[+15.99492]GGNVVIFDTVITNQEEPYQN[+2715.96255]HSGR.F	HexNAc(5)Hex(6)Fuc(1)NeuAc(2)		
R.NPPM[+15.99492]GGNVVIFDTVITNQEEPYQN[+2351.85075]HSGR.F	HexNAc(4)Hex(5)Fuc(3)NeuAc(1)		
R.NPPM[+15.99492]GGNVVIFDTVITNQEEPYQN[+2278.80922]HSGR.F	HexNAc(5)Hex(6)NeuAc(1)		
R.NPPM[+15.99492]GGNVVIFDTVITNQEEPYQN[+2205.79284]HSGR.F	HexNAc(4)Hex(5)Fuc(2)NeuAc(1)		
R.NPPM[+15.99492]GGNVVIFDTVITNQEEPYQN[+2204.77244]HSGR.F	HexNAc(4)Hex(5)NeuAc(2)		
R.NPPM[+15.99492]GGNVVIFDTVITNQEEPYQN[+2076.75025]HSGR.F	HexNAc(4)Hex(6)Fuc(2)		
R.NPPM[+15.99492]GGNVVIFDTVITNQEEPYQN[+2059.73493]HSGR.F	HexNAc(4)Hex(5)Fuc(1)NeuAc(1)		
R.NPPM[+15.99492]GGNVVIFDTVITNQEEPYQN[+1768.63952]HSGR.F	HexNAc(4)Hex(5)Fuc(1)		

Note: left two panels: glycopeptides and glycans detected in C1q without PG0352 treatment; right two panels: glycopeptides and glycans detected in C1q treated with PG0352.
