# Supplementary figures and images for: Functional and structural analyses reveal that a dual domain sialidase protects bacteria from complement killing through desialylation of complement factors
Source: PLoS Pathog. 2023 Sep 25;19(9):e1011674. doi: 10.1371/journal.ppat.1011674 (PMC10553830; doi:10.1371/journal.ppat.1011674)

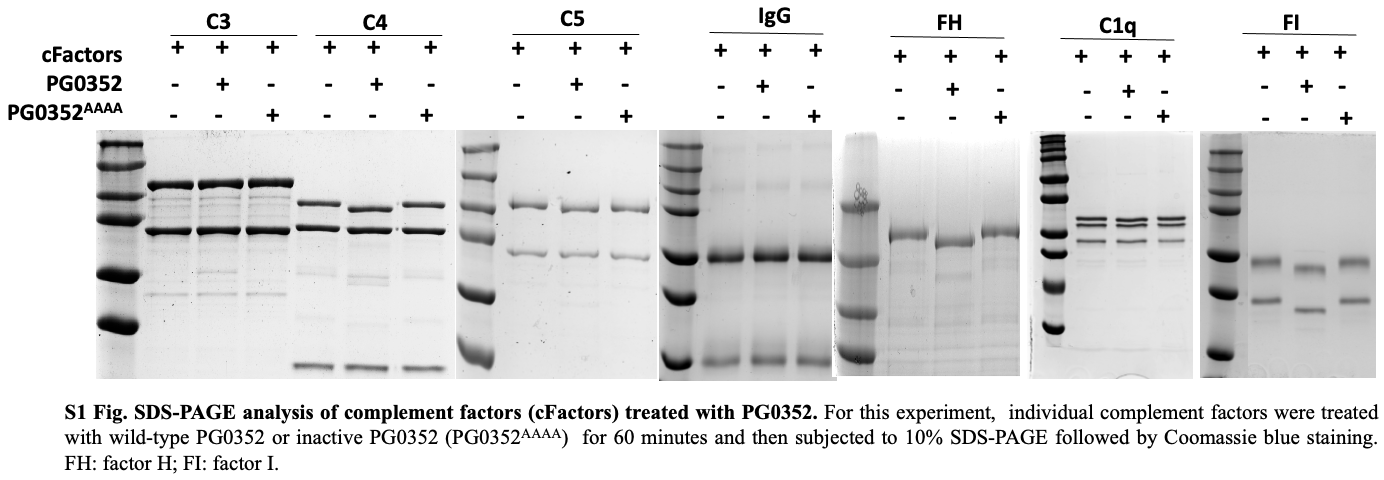

Supplement: S1 Fig — For this experiment, individual complement factors were treated with wild-type PG0352 or inactive PG0352 (PG0352AAAA) for 60 minutes and then subjected to 10% SDS-PAGE followed by Coomassie blue staining. FH: factor H; FI: factor I. (TIFF) [file ppat.1011674.s004.tiff]

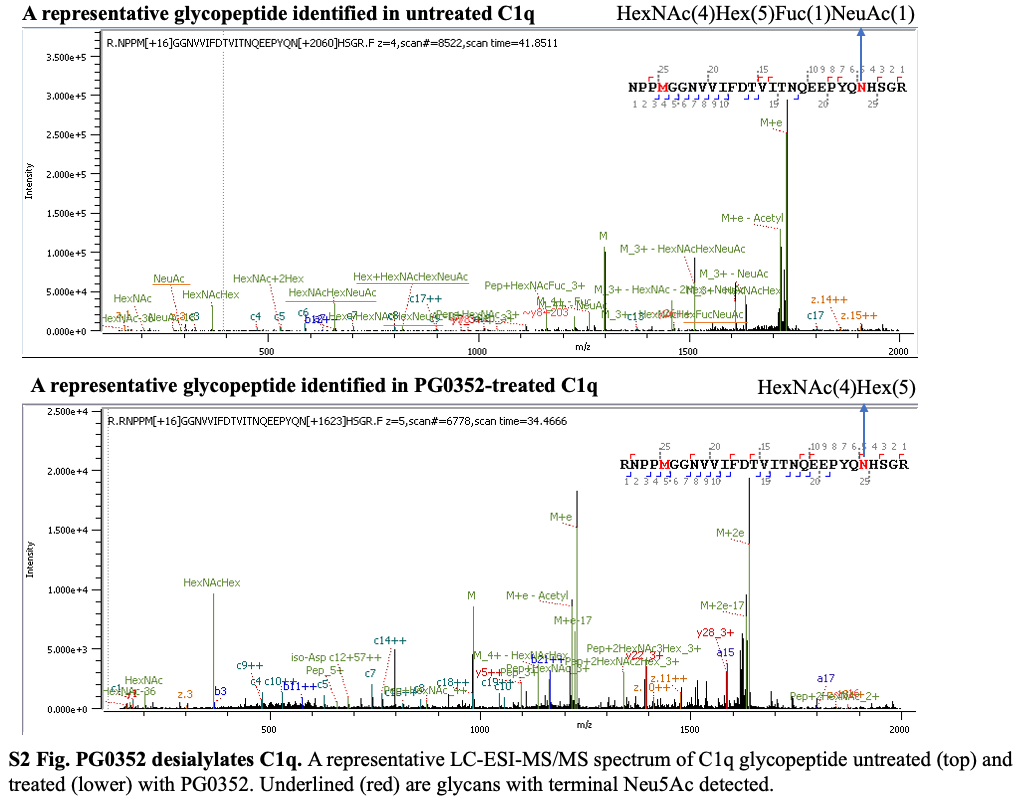

Supplement: S2 Fig — A representative LC-ESI-MS/MS spectrum of C1q glycopeptide untreated (top) and treated (lower) with PG0352. Underlined (red) are glycans with terminal Neu5Ac detected. (TIFF) [file ppat.1011674.s005.tiff]

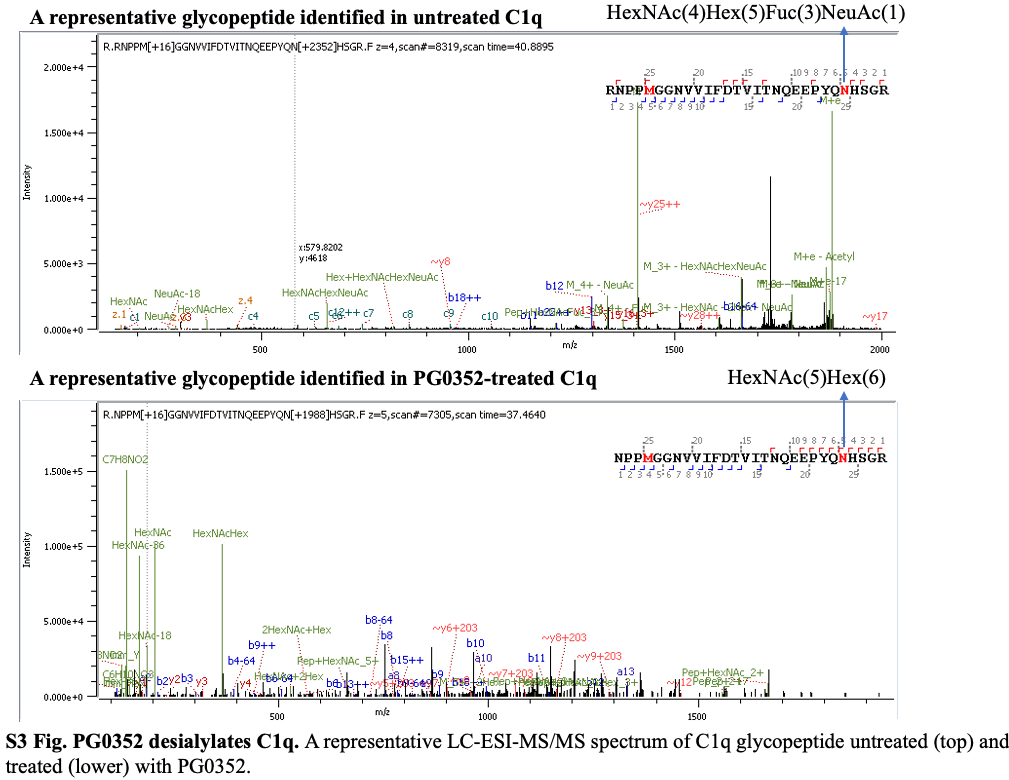

Supplement: S3 Fig — A representative LC-ESI-MS/MS spectrum of C1q glycopeptide untreated (top) and treated (lower) with PG0352. (TIFF) [file ppat.1011674.s006.tiff]

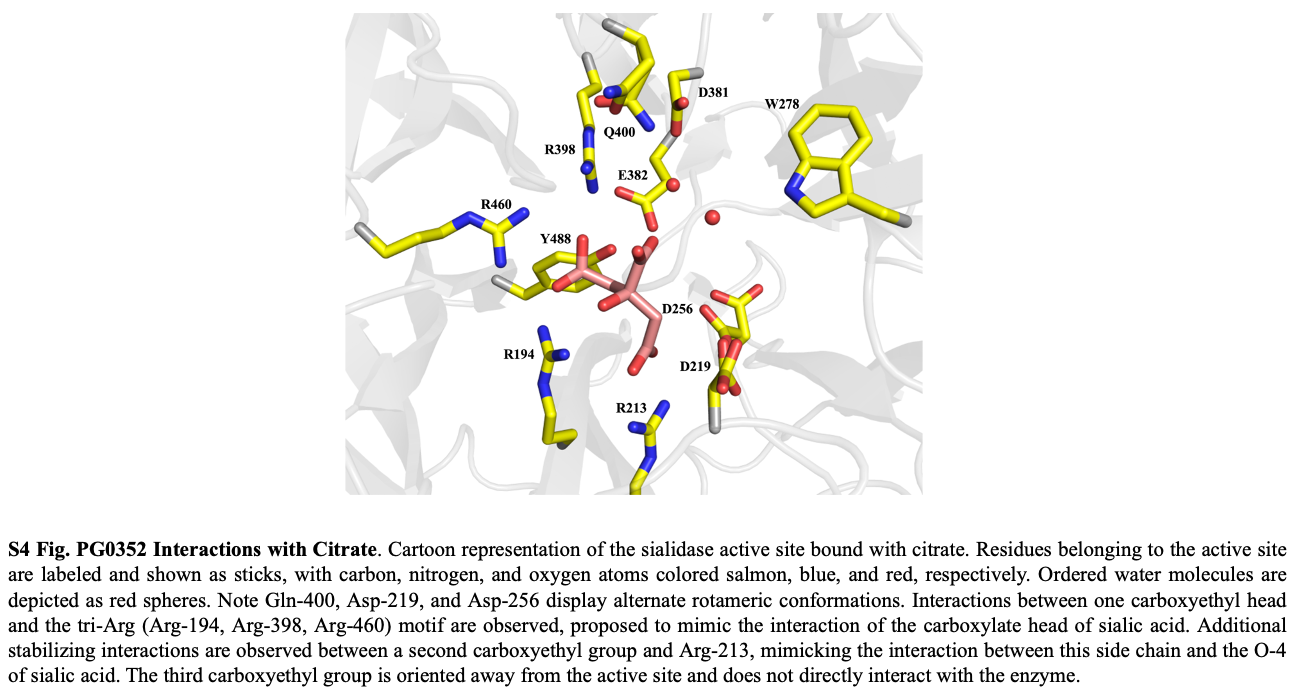

Supplement: S4 Fig — Cartoon representation of the sialidase active site bound with citrate. Residues belonging to the active site are labeled and shown as sticks, with carbon, nitrogen, and oxygen atoms colored salmon, blue, and red, respectively. Ordered water molecules are depicted as red spheres. Note Gln-400, Asp-219, and Asp-256 display alternate rotameric conformations. Interactions between one carboxyethyl head and the tri-Arg (Arg-194, Arg-398, Arg-460) motif are observed, proposed to mimic the interaction of the carboxylate head of sialic acid. Additional stabilizing interactions are observed between a second carboxyethyl group and Arg-213, mimicking the interaction between this side chain and the O-4 of sialic acid. The third carboxyethyl group is oriented away from the active site and does not directly interact with the enzyme. (TIFF) [file ppat.1011674.s007.tiff]

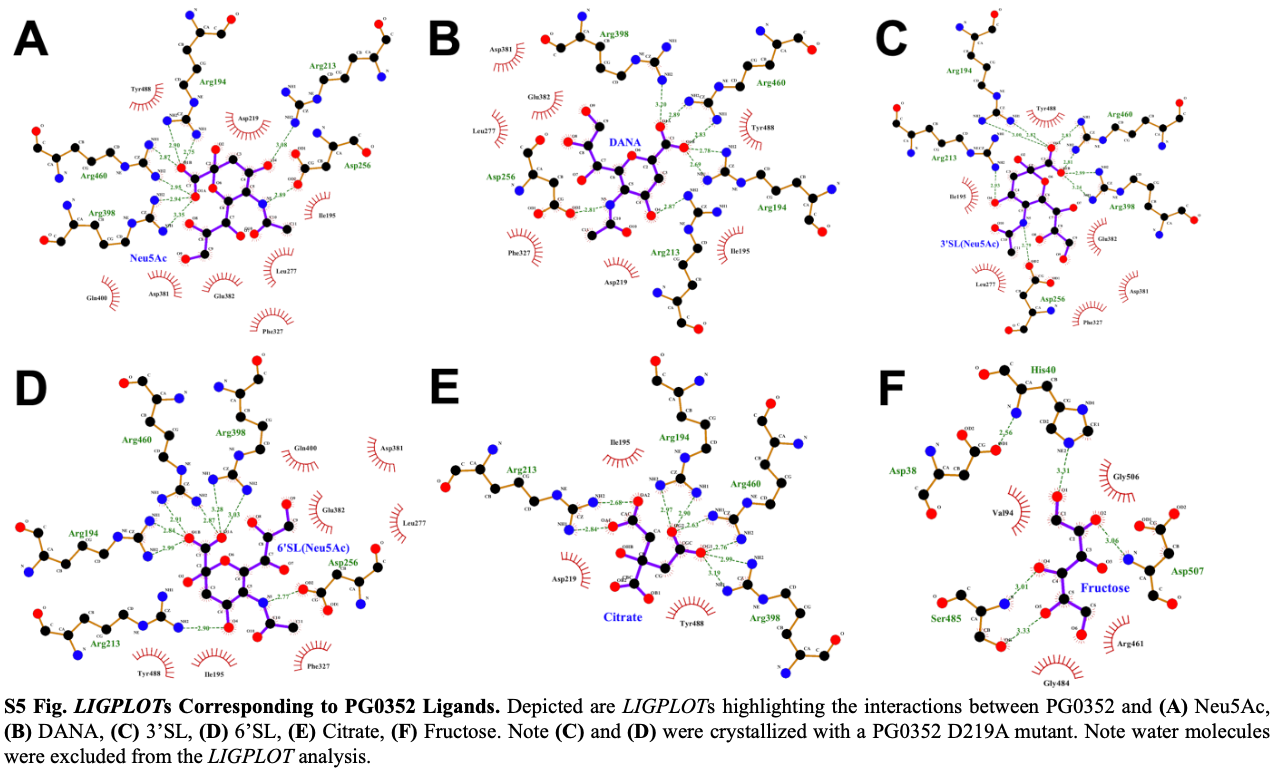

Supplement: S5 Fig — Depicted are LIGPLOTs highlighting the interactions between PG0352 and (A) Neu5Ac, (B) DANA, (C) 3’SL, (D) 6’SL, (E) Citrate, (F) Fructose. Note (C) and (D) were crystallized with a PG0352 D219A mutant. Note water molecules were excluded from the LIGPLOT analysis. (TIFF) [file ppat.1011674.s008.tiff]

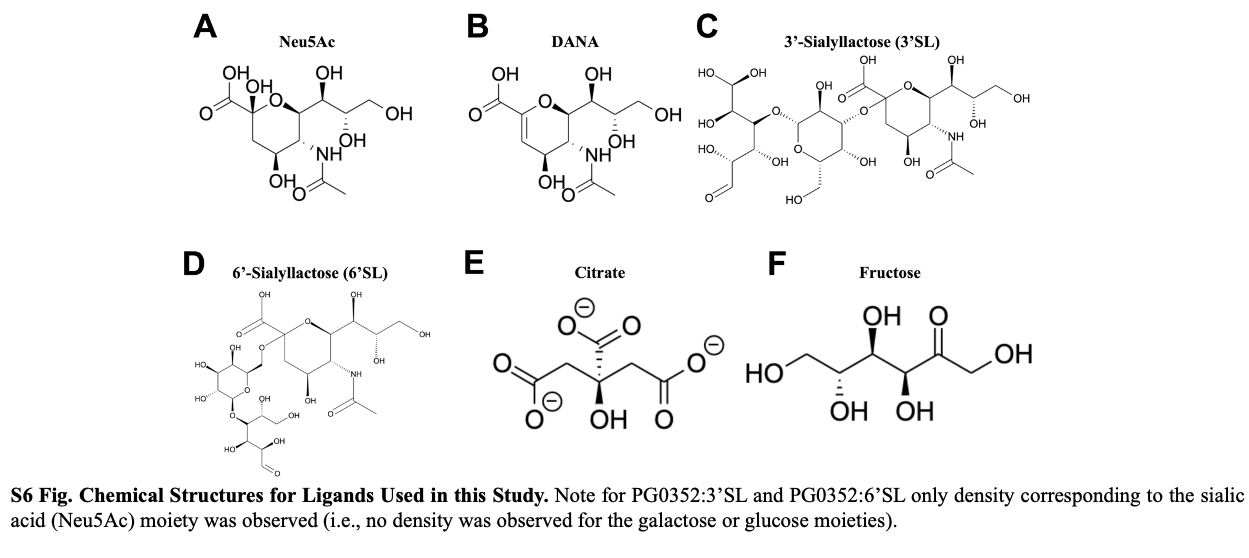

Supplement: S6 Fig — Note for PG0352:3’SL and PG0352:6’SL only density corresponding to the sialic acid (Neu5Ac) moiety was observed (i.e., no density was observed for the galactose or glucose moieties). (TIFF) [file ppat.1011674.s009.tiff]

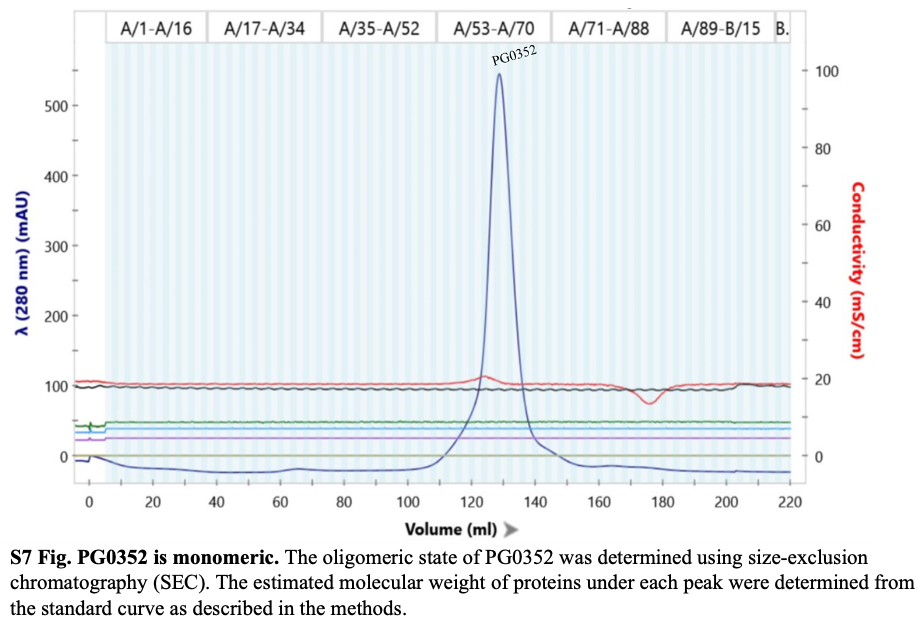

Supplement: S7 Fig — The oligomeric state of PG0352 was determined using size-exclusion chromatography (SEC). The estimated molecular weight of proteins under each peak were determined from the standard curve as described in the methods. (TIFF) [file ppat.1011674.s010.tiff]
