# Supplementary material for: Functional and structural analyses reveal that a dual domain sialidase protects bacteria from complement killing through desialylation of complement factors
Source: PLoS Pathog. 2023 Sep 25;19(9):e1011674. doi: 10.1371/journal.ppat.1011674 (PMC10553830; doi:10.1371/journal.ppat.1011674)
Supplement: Attachment — Submitted filename: PPathogens-D-23-01106R1-Point-by-point responses.docx [file ppat.1011674.s012.docx]

**Part II – Major Issues: Key Experiments Required for Acceptance**

**Reviewer #1:** There are no major issues identified.

**Reviewer #2:**

*1. Although several complement proteins are subject to the sialidase activity of recombinant PG0352, the biologically relevant target(s) of this activity is (are) not identified. De-sialylation of several complement proteins has been investigated; in some cases, e.g. C1q and C1 inhibitor, there is no or little effect (https://pubmed.ncbi. nlm.nih.gov/6608959/,* [*https://pubmed.ncbi.nlm.nih.gov/1468580/*](https://pubmed.ncbi.nlm.nih.gov/1468580/)*), in other cases (e.g. FH), activity has been reported to increase (*[*https://pubmed.ncbi.nlm.nih.gov/6238095/*](https://pubmed.ncbi.nlm.nih.gov/6238095/)*) or decrease (*[*https://pubmed.ncbi.nlm.nih.gov/ 33777036/*](https://pubmed.ncbi.nlm.nih.gov/%2033777036/)*). Indeed, although it seems perfectly plausible that the biological target is one or more of the complement proteins the authors identify, it remains possible that some other mechanism is at work.*

**Response:** We carefully read these four papers cited by the reviewer. For the case of C1q (PMID: 6608959) and C1 inhibitor (PMID:1468580), due to technique limitations, these two papers did not thoroughly examine the sialyation of C1q and C1 inhibitor (e.g., mapping the glycosylation sites using MS/MS as we did) before and after sialidase treatment; thus, it is possible that de-sialylation is incomplete. Additionally, neither these two papers carried out complement killing assays to assess the impact of de-sialyation of C1q and C1 inhibitor on complement activation and its bactericidal activity. In the cases of FH, as the reviewer noticed, the conclusions from these two papers are inconsistent. Once again, this discrepancy could be due to technique limitations. In contrast to these four papers, in this report, we provide both functional and structural evidence that bacterial sialidases can disarm the complement system via de-sialylation of key complement factors. Specifically, we mapped the glycosylation sites of C1q and FH before and after the treatment with PG0352 using Nano LC-ESI-MS/MS (**Table 2, Fig 3, and Fig S1,2**). Our results unequivocally demonstrate that the terminal sialic acid in C1q and FH are completely removed by PG0352.

In addition to the mechanism we report here, it is possible that other mechanisms are at work as discussed in the manuscript (**line 442-451**). For example, *Neisseria gonorrhoeae* evades complement killing by modifying its surface lipooligosaccharides (LOS) with sialic acids to capture host FH (PMID:26630657), a negative regulator of complement system. A similar scenario may exist in *P. gingivalis* because its LPS are sialylated, e.g., deletion of PG0352 may impair LPS sialylation which in turn affects *P. gingivalis* resistance to the complement killing. Additionally, gingipains, a group of proteases of *P. gingivalis*, cleave complement proteins. Aruni et al reported that deletion of PG0352 reduces the activity of gingipains (PMID: 21502589). Thus, it is also possible that PG0352 contributes to serum resistance by controlling the activity of gingipains.

*2. Although the sialidase activity is required for de-sialylation of complement proteins by recombinant protein, the role of this activity in Pg is unexplored; a Pg mutant producing enzymatically inactive PG0352 is not characterized.*

**Response:** In our previous study (PMID: 22025518), we constructed a deletion mutant of PG0352 and found that this mutant fails to assemble intact capsules, forms less biofilms, becomes susceptible to complement killing, and is less virulent in mice. Other groups also report that deletion of PG0352 impairs *Pg* gingipain activity,  attachment and invasion of oral epithelial cells, and its ability of inducing pro-inflammation responses (PMID: 31517596, 29675399, 28362225, and 21502589). Collectively, these reports highlight the importance of PG0352 in the pathophysiology of *P. gingivalis*. In this report, we seek to understand the biochemical and structural features of PG0352 and to elucidate the role of PG0352 in complement resistance and its underlying mechanism.

**Reviewer #3:** There are no major concerns.

**Part III – Minor Issues: Editorial and Data Presentation Modifications**

**Reviewer #1:**

*1. Complement proteins are present in the blood. Thus, complement evasion often occurs when pathogens come in contact with blood. Pg is often localized at the cavity of teeth. Occasionally, Pg may disseminate systematically. Therefore, would the PG0352-mediated complement evasion mostly occur during the systemic infection of Pg? The implications of the findings would need to be discussed.*

**Response:** As a keystone periodontal pathogen, *P. gingivalis* primarily inhabits the gingival crevices. The major component of crevicular ﬂuid is plasma which contains various sialylated proteins and active complement system. Therefore, the PG0352-mediated complement evasion primarily occurs at the gingival crevices. Of course, this evasion can also occur during the systemic infection of *Pg*. As suggested, this implication is discussed (**line 379-400**)

*2. Line 52-53, This manuscript is about the mechanisms of Pg in evading complement. Therefore, the C3 inhibitors as therapeutics to treat periodontitis may be not relevant to this work and may need to be removed.*

**Response:** Agreed, and changed as suggested.

*3. Lin 99, OspE accounts for a group of proteins, and each Lyme borrelia species or strain differs in the number of copies of ospE family it encodes. That is, B. burgdorferi strains may have different number of CRASPs. Therefore, it is better to remove "three" in line 99.*

**Response:** Agreed, and changed as suggested.

*4. The findings revealed by the newly solved high resolution structure of PG0352 is important. Such findings may be better to be summarized by a sentence and added at the end of the intro.*

**Response:** We truly appreciate this suggestion and changed it accordingly.

**Reviewer #2:**

*1. The polyalanine mutant PG0352AAAA was included in the experiments of Figs. 1, 2, and 4A and B, but not Figs. 3 and 4C. It would be useful to include this control throughout.*

**Response:** we created this inactive form of enzyme and used it as a negative control to rule out the possibility of protease contamination and potential impact of PG0352 on targeted protein stability. The experiment shown in Fig 3 was repeated 3 times with/without a control of PG0352^AAAA^. Per the reviewer’s suggestion, a new figure with the control is provided. Fig 4C did not include the control of PG0352^AAAA^ because Fig 4A & B already showed that this inactive enzyme has no protection on *E. coli*.

*2. The authors have shown that PG0352 removes sialic acid from complement proteins and immunoglobins using various assays systems in Figs.1-4. Have any experiments been done to test whether PG0352 desialylates other proteins in plasma?*

**Response:** Since this report focuses on the complement system, we only examined key complement factors that are known to be sialylated. Nevertheless, we did test the desialylation activity of PG0352 on human serum. Our results showed that PG0352 can desialylate human serum proteins in a dose- and time-dependent manner as shown in Fig 1.

*3. Have the authors determined if human serum contains antibody that recognizes PG0352?*

**Response:** No, we have not. The human serum samples used in this report were collected from healthy individuals who have no periodontitis.

*4. Figure 2A-H. A. Full-length PG0352 desialylates multiple complement proteins including C4, C5, FH and C4BP. However, in Fig 8D, many of these proteins were not identified in the pull-down assay using CBM. Are there any experiments, possibly using another assay system, that tests full length (and CBM) PG0352 for binding to C4, C5, FH and C4BP? B. The bands for the marker cannot be seen; the authors can just label bands to the left of the blot. C. A degradation control for Fig. 2, like that performed for Fig. 1, should be included.*

**Response: A.** In Fig 2A-H, purified complement proteins were used. In Fig 8D, human serum was used for the pull-down experiment. It is technically challenging and unnecessary to pull down and identify every individual sialylated proteins in the serum. To validate Fig 8D, we repeated pull-down assays using C1q, and our result showed C1q, but not the PG0352-treated C1q, was precipitated by rCBM-resin (Fig 8E), indicating that the binding between C1q and rCBM is specific. This conclusion is further substantiated by co-crystallization as shown in Fig 8F. Collectively, these three experiments clearly demonstrate that CBM functions as a carbohydrate-binding domain. **B.** As suggested, the sizes of molecular markers were added. **C.** While we did not include degradation control for Fig 2, we included the inactive enzyme PG0352^AAAA^ as a negative control to determine the observed desialylation is specific due to sialidase activity. In addition, the stability of treated-proteins was assessed by SDS-PAGE and Coomassie blue staining (**Fig S1**)

*5. Fig. 3B-C, the font is difficult to read.*

**Response:** A new figure is provided.

*6. Fig. 4C shows a decrease in complement deposition when E. coli is treated with PG0352. The bands in the Coomassie gel of the cell lysate are faint for both conditions and the authors should consider densitometric analysis.*

**Response:** Since many protein bands in the Coomassie gel are either too weak or too close to be quantified by densitometric analysis, we conducted immunoblotting analysis using GroEL as a loading control to show a same amount of *E. coli* samples was loaded. Notably, for this experiment, a defined amount of *E. coli* cells (10^7^) was used.

*7. In the text, the authors provide a thorough description of key interactions between the sialidase domain and its substrates in Fig 6A-D. However, identification of key interactions in these figures, as well as in Fig 8F, should be included. In addition, a LIGPLOT in the supplemental information would be a great addition as this would provide a visualization of the detailed interactions of key residues.*

**Response:** We have modified the Fig 6 legend to include details regarding the key interactions between the sialidase domain and substrates: coordination of the carboxylate head of sialic acid is mediated by the Tri-Arg motif (Arg194, Arg398, Arg460). The glycerol moiety is stabilized by interactions with Asp381, while the N-acetyl moiety is stabilized by polar interactions with Asp256 and insertion into a hydrophobic pocket formed by Leu220, Val272, Leu277, Trp278, and Phe327 (**line 1077-1080**). We did not make significant changes to Fig 8F as the legend points out that the depicted residues interact with the bound fructose but have modified the text to read “Residues making key interactions with fructose…”. As suggested, we added *LIGPLOTS* for each of the bound ligands (Neu5Ac, DANA, 3’SL, 6’SL, and fructose) (**Fig S5**).

*8. Line 112. Clarify what is meant by "molecular mechanism". Mechanism of cleavage? Downstream effect of cleavage?*

**Response:** Agreed, and revised as suggested.

*9. Line 136: “PG0352 construct in which residues Tyr-193….” Please clarify why were these residues chosen, e.g., a reference that highlights the importance of these residues. Does the mutant fold normally, e.g., by circular dichroism?*

**Response:** Please refer to the response to Q4 from Reviewer 3.

*10. Line 184-185 and Fig.3. Have any studies addressed the activity of the truncated protein (PG0352CT) in di-sialylation of FH or C1q?*

**Response:** We have tried to address this question; however, this truncated protein is insoluble. As shown in Fig 8A, despite of using 4 liters of *E. coli* cultures, only a small amount of soluble PG0352CT was purified. Filter paper spot assays showed this truncated protein still has sialidase activity, but its amount and purity are insufficient for us to perform further biochemical analysis.

*11. In Line 232 and 233, the authors state RMSD values of 2.1 and 1.54 as exhibiting “strong structural homology”, could the authors clarify the statements? Was this compared to structures with RMSD values greater than 2.1?*

**Response:** We have modified the text in line 233 and removed the word “strong”. The text now reads, “*Tf*NanH also exhibits structural homology to PG0352, with an RMSD of 1.54Å for 318 equivalent Cα atoms, despite only sharing 21% sequence identity within this domain.” Traditionally, RMSD values ≲ 2Å are considered structurally homologous. Comparison of structures with RMSD values greater than 2.1Å were not conducted, as these structures would have little structural similarity to PG0352. However, most GH33 sialidases display the conserved features outlined in lines 256-267: “The residues involved in catalysis and the sequence motifs that contribute to shaping the structure of the sialidase domain for GH33 enzymes are completely conserved in PG0352. There are four Asp-box motifs located on the opposite face of the active site that serve to maintain the β-propeller fold architecture of the enzyme… The positions of the side chains of Tyr-488 and Glu-382 suggest that they serve as the nucleophile and the general base catalyst, respectively, as they occupy topologically equivalent positions in other structurally characterized bacterial sialidase enzymes. Similarly, Asp-219 is positioned to function as the acid/base catalyst to activate a water molecule for release of free sialic acid.”.

*12. Lines 246-255, describes in detail of the interaction between citrate and PG0352, but the data is not shown. Could a figure of this data be presented in the supplemental?*

**Response:** We have added a figure showing citrate bound in the active site of PG0352 and the corresponding *Ligplot*, Fig. S4 and S5E, respectively.

*13. Fig. 8A, 8D and 8E. Label the molecular weights of reference bands on the lane of protein markers; Fig. 8D and 8E vs. Fig. 8 legend. “CMB” in the figures might be changed to “rCMB” to make consistent with legend.*

**Response:** Thanks, and revised as suggested.

*14. Line 132-134, The authors used Sambucus nigra (SNA) lectin to specifically detect α-2,3- and α-2,6-linked sialic acids. Fig. 1 showed that several protein bands were detected in normal human serum treated with inactive PG0352 by SNA blot analysis. Are there other types of sialic acids in human serum, which can’t be recognized by SNA, but contribute to complement activation and can be de-sialylated by PG0352.*

**Response:** It is possible but it is technically challenging to address this question because human serum contains ample glycosylated proteins with terminal sialic acids and their glycosidic linkages are poorly characterized. Thus, we do not know if those sialylated proteins can be recognized by SNA or other lectins.

*15. Line 466. It is clear to any reader that the structural studies were performed independently of the study of ref. 47. It is my opinion that the results of ref. 47 should be mentioned earlier in the manuscript, e.g., in the Results section.*

**Response:** Agreed, and changed as suggested.

**Reviewer #3:**

*1. It will be helpful to provide some background how the authors came about PG0352. Which strain did you use?  At first read, I was not sure if PG0352 was a strain name or something else.*

**Response:** Agreed, and changed as suggested. The name of *P. gingivalis* strain was described.

*2.  The figure legends need more details. The full names of abbreviations in the figures and diagram should be spelled out in the legends, e.g., SNA, ConA, etc. The amounts  of proteins used should also be listed.  One should not need to read Methods to understand how the experiment was done.*

**Response:** Agreed, and those missing details were added to the figure legends.

*3. In Figure 1 & 2, SDS-PAGE or Western should be included to ensure the disappearing bands were not due to protease contaminations.*

**Response:** In Fig 1, we carried out both SDS-PAGE and immunoblotting analyses using ConA to rule out the possibility of protease contaminations. To save the space, we only included ConA western blots. Also, in both Fig 1 & 2, we included an inactive enzyme (PG0352^AAAA^) as a negative control to further rule out the possibility of protease contaminations. Furthermore, SDS-PAGE was applied to determine if the stability of those tested complement factors is affected (**Fig S1**).

*4. For mutant PG0352AAAA, what is the rationale to make Ala substitutions at these positions?  These positions, as well as other important residues, should be labeled in the structure in Figure 5.  The Neu5Ac coloring is not easy to discern--pink and red are difficult to differentiate.*

**Response:** We added rational for the poly-Ala substitution at residues 193-196 in **lines 136-138**. We modified Fig 5 to show the location of the conserved “Y/FRIP” motif and added a corresponding description in the figure legend. We did not include active site residues, as addition of these residues cluttered the figure and made it unpleasant to our eyes. Additionally, these residues are highlighted in Fig 6. We changed the coloring of the Neu5Ac in Fig 5 to green to make differentiation of the carbons, nitrogens, and oxygens easier.

*5. Figure 6 is confusing. Consider only highlighting differences between ligands, as well as the PG0352 residues.*

**Response:** Fig 6 included all four ligand-bound active sites to highlight the similarity of binding for Neu5Ac, DANA, 3’SL, and 6’SL. The only significant differences observed are in the interactions with Tyr-488 and Glu-382, as outlined in the text, and additional ordered water molecules observed upon DANA and 3’SL/6’SL binding. Fig 6 also serves to show the corresponding electron density for each of the ligands. Furthering the comparison of ligand binding, we added *LIGPLOTS* for each of the ligands, as requested by Reviewer 2 (**Fig S5**), and included chemical structures for each of the ligands (**Fig S6)**.
