# Supplementary material for: Functional and structural analyses reveal that a dual domain sialidase protects bacteria from complement killing through desialylation of complement factors
Source: PLoS Pathog. 2023 Sep 25;19(9):e1011674. doi: 10.1371/journal.ppat.1011674 (PMC10553830; doi:10.1371/journal.ppat.1011674)
Supplement: Attachment — Submitted filename: Letter-PPathogen-D-23-01106R1 .docx [file ppat.1011674.s013.docx]

September 8, 2023

**Re: PPATHOGENS-D-23-01106R1**

Dear Dr. Jon Skare and other editors,

Thank you again for overseeing this manuscript. Per your request, we addressed a single comment from Reviewer 2 and revised the manuscript accordingly. See below for the detail:

*“The authors should explain in the Results section leading up to Fig 4 the reasons why they cannot perform the experiment in PG rather than the E. coli surrogate. This might include issues of expression of the inactive mutant in PG or the inability to perform "extracellular" complementation by adding recombinant protein to PG prior to challenging with complement. Then, in the Discussion, the authors should comment on the unavoidable limitations of utilizing the E. coli surrogate. These additions will make it clear to readers that the authors have taken a systematic approach but one limited by technical challenges beyond their current control.”*

**Response:** We truly appreciate this suggestion and revised as suggested. See details in Line 191-198 and Line 435-440.

We have addressed all the concerns raised by the reviewers and hope that this revision is now suitable for publication in *PLoS Pathogens.*

Yours Sincerely,

Chunhao (Chris) Li

Eddie Myers Endowed Professor

Oral and Craniofacial Molecular Biology

Microbiology and Immunology

Virginia College of Medicine

Virginia Commonwealth University
